# A Globally Distributed Cyanobacterial Nitroreductase Capable of Conferring Biodegradation of Chloramphenicol

**DOI:** 10.34133/research.0692

**Published:** 2025-05-08

**Authors:** Qiu-Lian Zhong, Jiu-Qiang Xiong

**Affiliations:** ^1^ College of Marine Life Sciences, Ocean University of China, Qingdao, Shandong, China.; ^2^ Anhui Provincial Key Laboratory of Environmental Pollution Control and Resource Reuse, Anhui Jianzhu University, Hefei 230601, China.

## Abstract

Cyanobacteria play pivotal roles in global biogeochemical cycles and aquatic ecosystems due to their widespread distribution and significant contributions to primary production. Yet, the interactions between cyanobacteria and antibiotics remain unclear. This study revealed that *Synechocystis* sp., a cyanobacterial species, removed 94.27% of 0.1 mg l^−1^ chloramphenicol (CAP) through enzyme-mediated degradation. While cytochrome P450 enzymes (CYP450s) were found unnecessary for CAP removal, a gene encoding cyanobacterial nitroreductase was significantly up-regulated (7.85-fold) under CAP exposure. The purified nitroreductase exhibited strong binding affinity to CAP (*K*_d_ = 2.9 nM) and a Michaelis constant (*K*_m_) of 104.0 μM. By engineering a bacterial strain with nitroreductase, 94.43% of 0.1 mg l^−1^ CAP was removed within 2 h. Metagenomic and metatranscriptomic analyses showed that nitroreductase genes and transcripts are globally distributed across diverse microbial phyla. These findings uncover a novel enzyme for CAP degradation and advance sustainable biotechnologies to mitigate antibiotic pollution, addressing critical environmental challenges in aquaculture and other industries globally.

## Introduction

Aquaculture activities provide proteins for 35% of global population, which accounts for at least 20% of all the world’s protein sources [1]. Growing populations and economy enhance such demands for animal-derived food products, which drive massive livestock/aquaculture cultivation at various industrial scales [2,3]. This practice causes extensive use of water resources and antibiotics to promote the health and growth of animals [4,5]. For example, it is reported that 6.6 × 10^10^ m^3^ of water was used for pond aquaculture in 2018 in China [6]. Global antibiotic usage per ton of animal biomass ranges from 0.04 to 0.91 kg [7]. Unfortunately, 50% to 70% of uptaken drugs can be discharged with excretions of animal urines and feces, which were directly discharged along with the aquaculture wastewater into environments. This not only causes frequent distribution of diverse antibiotics [8] but also spreads antibiotic-resistant genes through a global water cycle [9]. Thus, the rapid expansion of aquaculture has led to concerns regarding its environmental footprint, particularly the discharge of wastewater containing antibiotics.

Aquaculture wastewater is rich in nitrogen, phosphate, and chemical oxygen demand, making it conducive to the growth of microalgae. Cyanobacteria play pivotal roles in global biogeochemical cycles and aquatic ecosystems due to their widespread distribution and significant contributions to primary production [10,11]. Researchers have tried to apply microalgae-based biotechnologies for removal of frequently found antibiotics, and concluded that microalgae can achieve >90% removals of these chemicals [12,13]. Real-scale investigation demonstrated that microalgae can achieve over 90% removal of antibiotics from wastewater, which is more effective than conventional methods like sludge processing [12]. Additionally, microalgal biomass can be used for generation of high-value-added byproducts or as food sources for aquaculture [14]. Thus, microalgae-based biotechnologies have been one of the most attractive strategies to treat antibiotic-polluted aquaculture wastewaters [15,16]. However, how these microalgal species metabolize such persistent pollutants remains largely unexplored, which has caused lacking regulation strategies to increase its engineering feasibility.

Existing studies point out that the effectiveness of microalgae in degrading antibiotics is attributed to enzymatic reactions induced by cytochrome P450 (CYP450) and glutathione *S*-transferase since humans mainly metabolize antibiotics using these 2 kinds of enzymes [17,18]. However, there is no direct evidence linking the catalytic activities of these enzymes with the removal efficiencies/biotransformation of antibiotics [19]. Genes encoding CYP450s in the genomes of *Chlorella* and *Chlamydomonas* have been proven [18]. Unexpectedly, there was only 10% to 40% removal of persistent antibiotics such as sulfadiazine, sulfamethoxazole, norfloxacin, and amoxicillin in the *Chlorella* and *Chlamydomonas* remediation systems [20–22]. Such results indicate that there should be unknown or new enzymes mediating the degradation of antibiotics in microalgal cells.

Therefore, we conducted this study to explore whether CYP450s are necessary for degradation of antibiotics by performing the inhibiting experiments with taking chloramphenicol (CAP) and *Synechocystis* sp. as the test chemical and microorganism. CAP is a broad-spectrum antibiotic that has been widely accepted for usage in developing countries. Asia produces 90% of worldwide aquaculture food, and this volume will be double by 2050 [23]. Such data might indicate that CAP can be extensively used in Asia aquaculture. It is also true that CAP has been frequently found in surface waters, wastewaters, groundwaters, and sea waters in Asia countries [24–26]. For example, it was found that the residual concentration of CAP in wastewater and seawater can reach 47.4 and 15.6 μg l^−1^ [27,28]. Intriguingly, we found that CYP450s are not necessary for degradation of CAP in *Synechocystis* sp. system. We then identified what kind of potential enzymes is for the metabolism of antibiotics using RNA sequencing, protein heterologous expression, high-resolution mass spectrometry, molecular docking, and binding affinity analysis. As a concept of proof, we also investigated the global transcripts of the gene encoding target enzyme to see whether it is a conserved pathway in environment. Subsequently, we engineered a bacterial species for efficient treatment of antibiotics in 2 h. These findings reveal a novel enzyme capable of degrading CAP, advancing sustainable biotechnological solutions to combat antibiotic pollution and addressing pressing environmental challenges in aquaculture and other industries worldwide.

## Results and Discussion

### *Synechocystis* sp. tolerance capacity assessment

**Fig. 1.**
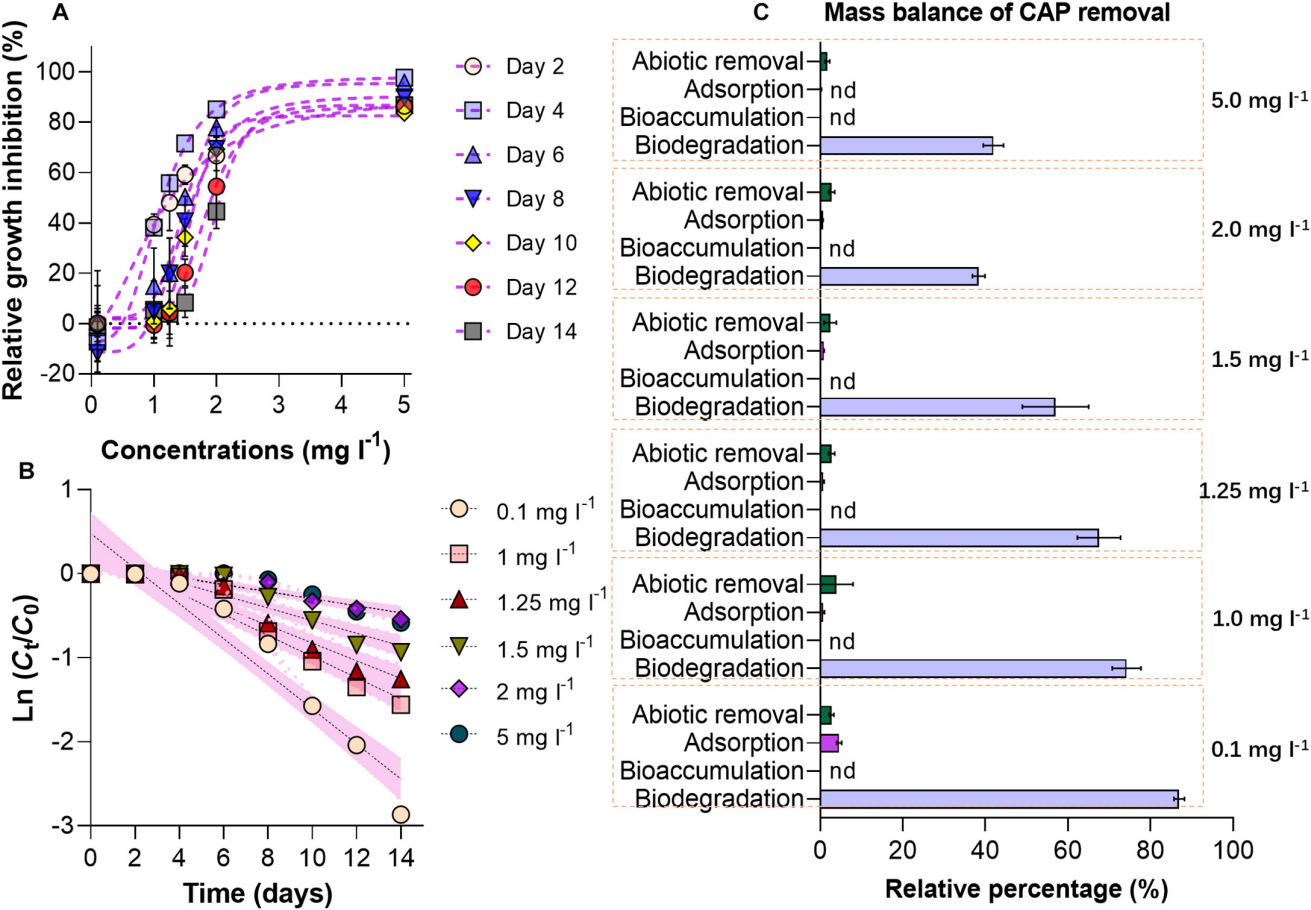
Tolerance and degradation capacity assessment of *Synechocystis* sp. (A) Relative growth inhibition of *Synechocystis* sp. induced by different concentrations (0.5, 1, 1.25, 1.5, 2, and 5 mg l^−1^) of CAP during 14 d of cultivation. A dose–response simulation model [inhibitor versus response–variable slope (4 parameters)] from GraphPad Prism 9 was used to analyze the data. (B) Removal kinetics of different concentrations (0.5, 1, 1.25, 1.5, 2, and 5 mg l^−1^) of CAP during 14 d of cultivation. The pink color indicated the 95% confidence interval of the mean value. (C) Mass balance revealed relatively removed amounts of different concentrations of CAP by abiotic factors, adsorption, bioaccumulation, and biodegradation. All experiments have been conducted in triplicate (*N* = 3).

We evaluated the tolerance capacities of used *Synechocystis* sp. (Fig. 1A). It can be seen that most inhibitory effects occurred at day 4, and there was 38.12, 55.74, 71.63, 85.09, and 97.66% reduction in the microalgal growth after exposure to 1.0, 1.25, 1.5, 2, and 5 mg l^−1^ CAP. However, the inhibitory effect gradually decreased with increasing cultivation duration, and subsequently, there was no significant effect of <1.25 mg l^−1^ CAP on *Synechocystis* sp. after 14 d of cultivation. Higher concentrations including 2 and 5 mg l^−1^ CAP significantly (*P* < 0.05) caused 44.47% and 86.78% inhibition on the microalgal growth even at day 14. The growth inhibition data fitted a dose–response model named inhibitor versus response–variable slope (4 parameters) well (*R*^2^ = 0.92 to 0.99) (Table S2). Accordingly, the calculated half-maximum effective concentrations (EC_50_) ranged from 1.09 to 2.0 mg l^−1^ for CAP during 14 d of cultivation. These data were at the same levels (mg l^−1^) as earlier reported EC_50_ values of diverse microalgal species toward different antibiotics [29,30]. All these results demonstrated that *Synechocystis* sp. has a high tolerance capacity toward CAP, which may indicate that *Synechocystis* sp. can be applied for treatment of CAP-polluted wastewaters.

### Cyanobacterial metabolism efficiency of CAP

We then evaluated the removal efficiencies of different concentrations of CAP by *Synechocystis* sp. during cultivation (Fig. 1B). Intriguingly, *Synechocystis* sp. showed a final removal of 94.27%, 78.93%, 71.12%, 60.31%, 41.85%, and 44.06% of 0.1, 1, 1.25, 1.5, 2, and 5 mg l^−1^ CAP, respectively, after 14 d of cultivation. This removal percentage was significantly higher compared to previously reported data. For example, Lai et al. [31] found only 6.8% to 23.5% removal of CAP by *Chlorella pyrenoidosa*, *Isochrysis galbana*, and *Tetraselmis chui*. The removal data also fitted a first-order model well since the *R*^2^ values ranged from 0.90 to 0.97. Correspondingly, the removal kinetic constant (*k*, d^−1^) of CAP ranged from 0.060 to 0.277 d^−1^ for the concentrations of 0.1 to 5 mg l^−1^ CAP, and the half-lives (*T*_1/2_, d) decreased from 11.70 to 2.50 d (Table S3). To investigate how CAP elimination occurred in *Synechocystis* sp. remediation system, we further calculated the mass balance of the CAP removals by quantifying the removed amounts by abiotic factors, adsorption, accumulation, and biodegradation (Fig. 1C). Negligible amounts (<3.9%) of CAP were removed by abiotic factors, adsorption, and accumulation, while biodegradation accounted for 38.38% to 86.96% of the total CAP removal. Biodegradation has been demonstrated as the main mechanism by our own and others’ works previously [32–34]; however, how this process induced efficient removal of antibiotics remains unknown.

### Functional enzymes involved in CAP degradation

**Fig. 2.**
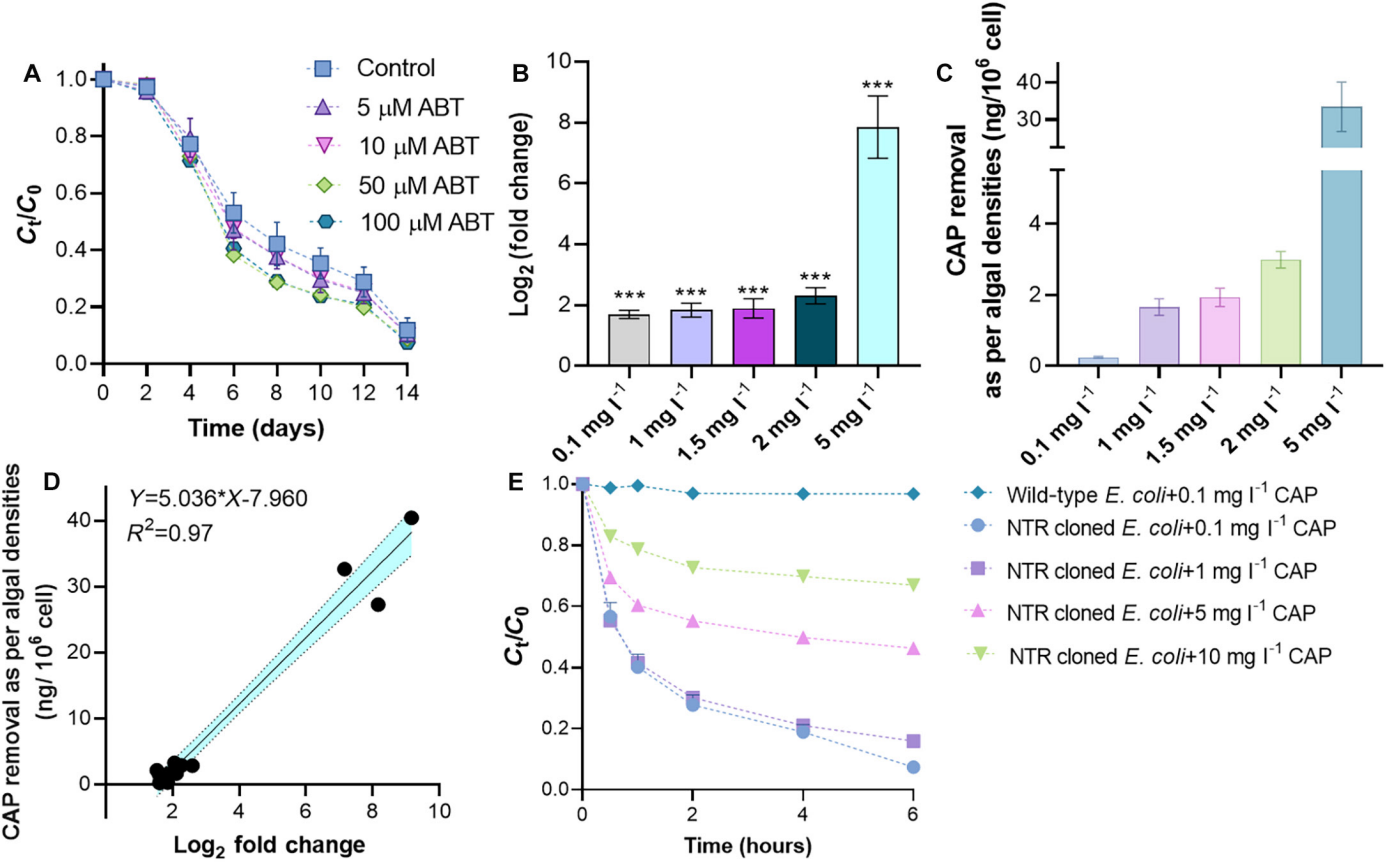
Verification of CYP450 activity and removal kinetics of CAP by an engineered bacterium carrying NTR gene. (A) Effects of different concentrations of a CYP450 enzyme inhibitor, ABT, on the removal of 0.1 mg l^−1^ CAP by *Synechocystis* sp. during 14 d of cultivation. (B) Log_2_ fold changes of the gene encoding *Synechocystis* sp. NTR induced by different concentrations (0.1, 1, 1.5, 2, and 5 mg l^−1^) of CAP. (C) Calculated amounts removed by 10^6^ *Synechocystis* sp. cells after 14 d of cultivation. (D) Correlation analyses between removed CAP (10^6^ *Synechocystis* sp. cells) and log_2_ fold change of *Synechocystis* NTR gene. (E) Removal kinetics of different concentrations of CAP without IPTG-induced *E. coli* carrying NTR gene [(+) vector] and *Synechocystis* NTR gene free (wild type) during 6 h of cultivation. Star symbols indicated significant differences among the treatment groups. All experiments have been conducted in triplicate (*N* = 3).

Most studies have claimed that CYP450s are responsible for the efficient degradation of diverse antibiotics [17,18]. We first evaluated the role of CYP450s by inhibiting experiments using 1-aminobenzotriazole (ABT) as an effective CYP450 inhibitor [35,36]. As shown in Fig. 2A, there was no significant difference in removal efficiency of CAP with or without ABT, which demonstrated that CYP450s in *Synechocystis* sp. does not determine the CAP removal rate. We speculated that other enzymes play essential roles, and identified potential functional enzymes by RNA-sequencing analyses and protein heterologous expression. As shown in Fig. 2B, we found that the log_2_ fold changes of a gene encoding nitroreductase (NTR) in *Synechocystis* sp. were significantly up-regulated by 1.70- to 7.85-fold compared to the control after exposure to different concentrations of CAP. Especially, the increased expression of NTR correlated with the removed amount of CAP by 10^6^ *Synechocystis* sp. cells under different CAP exposure groups. As shown in Fig. 2C, the removed CAP by 10^6^ cells was 0.24, 1.66, 1.93, 2.98, and 33.43 ng at 0.1, 1.0, 1.5, 2.0, and 5 mg l^−1^ CAP, respectively. This is consistent with the obviously elevated expression of NTR gene by 5 mg l^−1^ CAP. Meanwhile, the data showed that there was a significant linear relationship (*R*^2^ = 0.97) between the removed CAP (10^6^ cells) and the expression changes of gene encoding *Synechocystis* NTR (Fig. 2D). These results may indicate that NTR played essential roles in the removal of CAP by investigated *Synechocystis* sp.

**Fig. 3.**
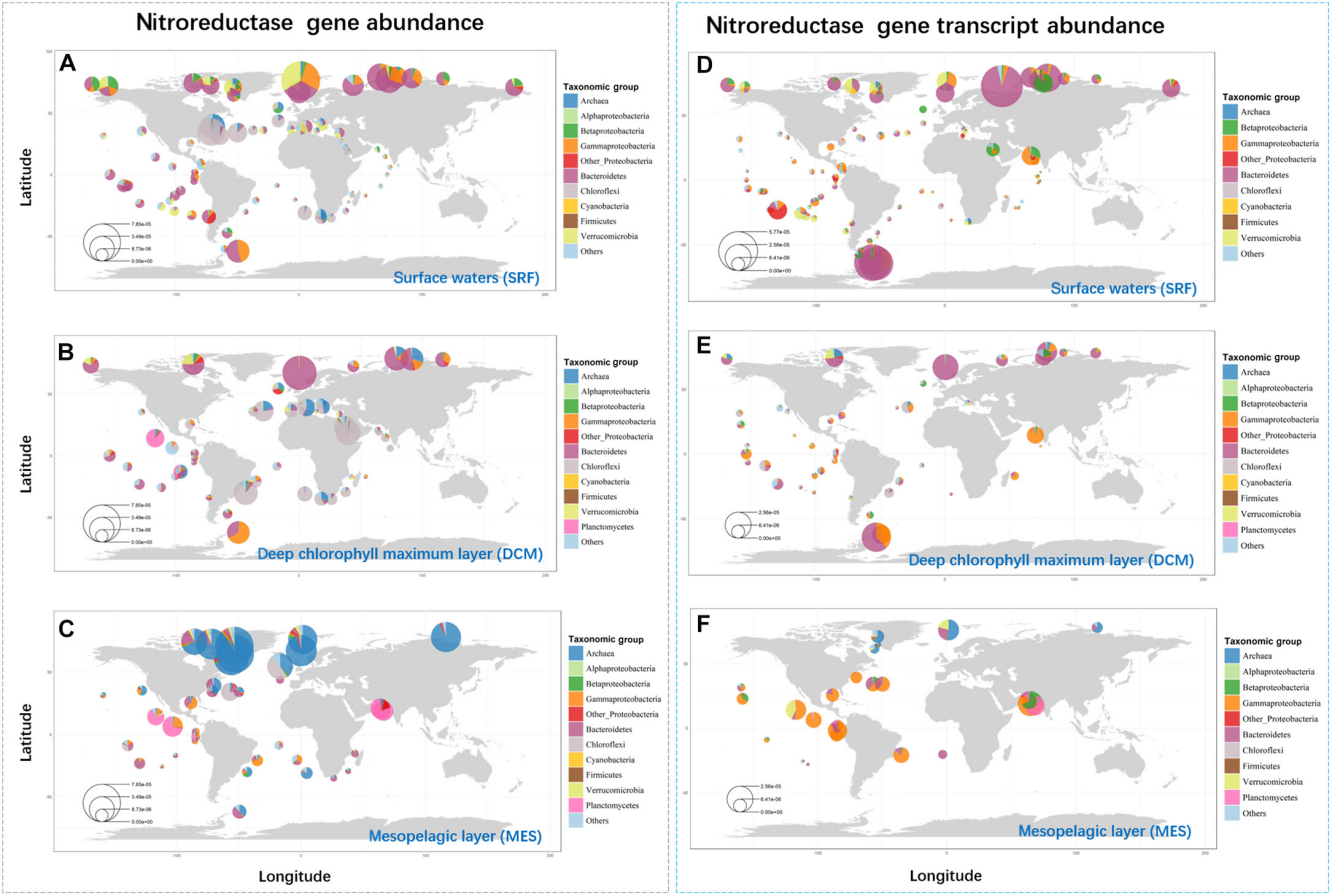
Global distribution of cyanobacterial NTR gene. (A to C) NTR gene abundance by taxonomic assignment in SRF, DCM, and MES of the global ocean. (D to F) NTR transcript abundance by taxonomic assignment in SRF, DCM, and MES. The circle radius represents normalized abundance in the Tara Ocean dataset, which was calculated as a percentage of the median gene abundance or transcript abundance of 10 single-copy marker genes. The relative abundance of different taxa at each site is shown as pie charts, summing taxonomy at the level of phylum. In the figure legend, unclassified is abbreviated to others for clarity of presentation. All data were derived using Tara Oceans metagenomes (OM_RGC_v2_metaG) and metatranscriptomes (OM_RGC_v2_metaT).

We then confirmed the removal efficiency of CAP by *Escherichia coli* with or without cloning NTR gene. As shown in Fig. 2E [without isopropyl β-d-1-thiogalactopyranoside (IPTG) induction], wild *E. coli* only removed 3.22% of 0.1 mg l^−1^ CAP in 6 h, while the results showed that *E. coli* carrying the *NTR* gene can efficiently (33.1% to 92.6%) remove CAP even without IPTG induction. We also investigated the removal kinetics of the same concentrations of CAP by *E. coli* carrying the *NTR* gene with IPTG induction (Fig. S3). *E. coli* carrying the *NTR* gene degraded 94.43% of 0.1 mg l^−1^ CAP within 2 h after IPTG induction. Meanwhile, *E. coli* carrying the *NTR* gene removed 91.5%, 72.9%, and 39.8% of 1, 5, and 10 mg l^−1^ CAP after 6 h of cultivation under the same experimental conditions. These data strongly suggested that *Synechocystis* sp. NTR can be used as a target biocatalyst for the treatment of CAP-polluted wastewater. While cyanobacteria are known to harbor diverse enzymatic capabilities including NTRs, the specific global occurrence of an NTR capable of degrading CAP would require targeted investigation and may vary widely depending on the ecological niche and genetic diversity of cyanobacteria in different regions.

### Distribution of identified *Synechocystis* sp. NTR

To investigate the distribution and transcriptional levels of cyanobacterial NTR genes, we utilized the OM-RGC_v2_metaG and OM-RGC_v2_metaT databases to obtain the corresponding abundance in global oceans [37]. We analyzed the metagenomic and metatranscriptomic data enriched for 0.22- to 3.0-μm size fractions associated with marine environments for homologs. Detailed information was provided in Tables S4 and S5, including NTR homologous genes, their taxonomic assignment, nucleic acid sequences, and protein sequences. Additionally, given the potential significant differences in community composition and species across different oceanic layers, we summarized the taxonomic affiliations, abundance, and environmental parameters of relevant genes and transcripts from the surface layer (SRF) to the intermediate depths of approximately 1,000 m [mesopelagic zone (MES)] (Tables S6 to S11).

Genes and transcripts within the 0.22- to 3.0-μm size fractions predominantly originated from prokaryotes, which suggested its ubiquitous occurrence in bacteria. As depicted in Fig. 3, the normalized gene and transcript abundances of NTR varied across the taxonomic group and the water depths. As shown in Fig. 3A to C, the NTR genes were widely distributed across the 3 oceanic water layers. The majority of marine NTR genes were closely related to Bacteroidetes, Archaea, Chloroflexi, and Gammaproteobacteria, while the NTR transcriptional activity was notably higher in the SRF. Nearshore marine areas have been primarily selected to conduct existing aquaculture [38]. Thus, it is reasonable to see frequent genes and transcripts of genes encoding CAP metabolic enzyme in the marine ecosystem. Within the taxonomic composition, the phylum Bacteroidetes contributed 63% of NTR transcripts in coastal Arctic and South Atlantic regions, followed by Gammaproteobacteria (13%) and Betaproteobacteria (8%) (Fig. 3D). The taxonomic contributions to NTR transcripts remained relatively stable in the deep chlorophyll maximum (DCM) layers, with Bacteroidetes at 50% and Gammaproteobacteria at 25% (Fig. 3E). These taxa are widely distributed in environments rich in organic matter, which are common in coastal and nearshore areas where aquaculture is prevalent. Such environments provide favorable conditions for bacteria that specialize in the breakdown and transformation of complex organic substrates, including antibiotics, which likely explains the dominance of Bacteroidetes and Gammaproteobacteria in these coastal regions [39,40]. The DCM layer, typically rich in primary production, is characterized by lower light availability but higher nutrient concentrations, especially in oligotrophic waters. The dominance of Bacteroidetes (50%) and Gammaproteobacteria (25%) in this layer suggests that these taxa are well adapted to the specific conditions of the DCM. Their ability to break down complex organic compounds enables them to thrive in nutrient-dense, organic matter-rich environments like the DCM, which serves as a hotspot for microbial activity and organic matter processing [39]. In contrast, the MES zone was dominated by Gammaproteobacteria (49%) in coastal Pacific and Arctic regions, although Archaea gene sequences were most abundant. This discrepancy may be because Archaea primarily inhabit extreme environments (such as high temperature, high salinity, high acidity, and hypoxia), which are not conducive to the function of NTR [41,42]. To conclude, NTR activity is particularly pronounced in Bacteroidetes and Gammaproteobacteria. This heightened activity may be attributed to their unique metabolic functions and ecological niches. Bacteroidetes and Gammaproteobacteria are widely distributed and abundant in organic matter-enriched environments, which play essential roles in the transformation of organic substrates including antibiotics [43]. The dominance of Bacteroidetes and Gammaproteobacteria in specific ecological zones, such as coastal and DCM layers, is likely driven by their metabolic capabilities to degrade complex organic compounds, including antibiotics, in environments rich in organic matter [40,44].

Based on the correlation analysis of environmental parameters, the abundance of the NTR gene was relatively high within a distance of 0 to 600 km from the coast, which may be related to the rich nutrient supply and high primary productivity in nearshore areas [45]. The presence of dissolved nutrient and organic matter enhanced the growth of microorganisms carrying the NTR gene, leading to its high abundance. The gene was also more prevalent under environmental conditions with an oxygen concentration of 175 to 400 μmol/kg and salinity levels of 32 to 40 PSU (practical salinity units). In addition, the primary distribution range of the NTR gene was within a pH of 7.7 to 8.1, indicating its preference for marine environments with near-neutral to slightly alkaline conditions. The broad temperature range (−1.64 to 30.59 °C) suggested that microorganisms harboring the NTR gene were well adapted to both cold and warm waters, reflecting their physiological adaptability (Fig. S4). The transcriptional activity of the NTR gene followed a distribution pattern similar to its abundance. Moreover, when nitrate/nitrite (NO₃^−^-NO₂^−^) concentrations ranged from 0 to 31 μM, total phosphorus (PO₄^3−^) concentrations from 0 to 3.2 μM, and iron concentrations from 0 to 0.0014 μM, the transcript abundance of the NTR gene seems at high levels (Fig. S5). The consistency between the spatial abundance and transcriptional activity of the NTR gene suggested that nitroreduction was a metabolically active and ecologically significant process in marine ecosystems. Its broad temperature adaptability highlighted the NTR gene’s potential role in sustaining microbial functions across diverse marine environments, which also further indicated that the NTR enzyme has promising potential for biotechnological and engineering applications.

### Functional strain construction and enzyme activity characterization

**Fig. 4.**
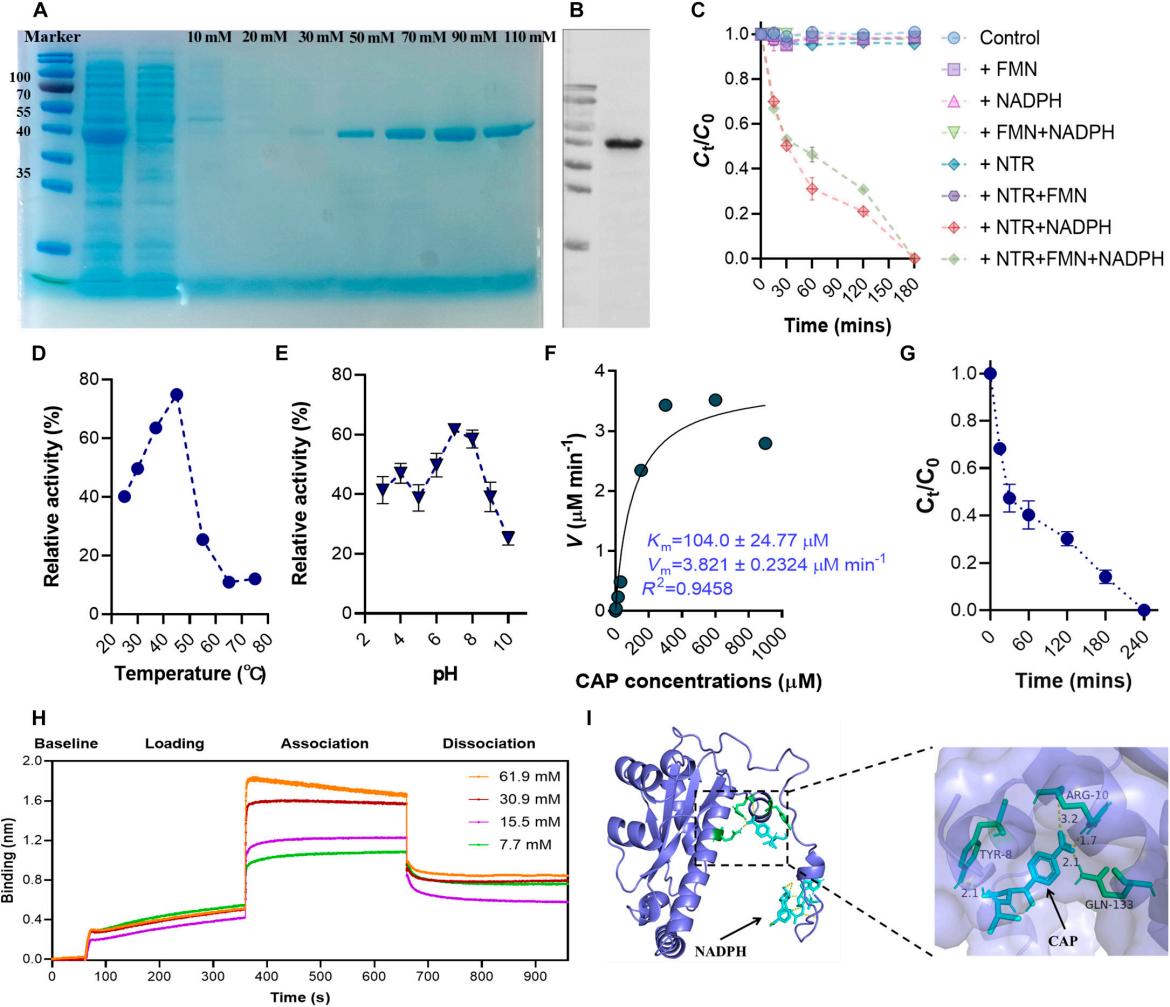
Functional characterization of purified NTR. (A) SDS-PAGE of eluted *Synechocystis* sp. NTR by a series of different concentrations of imidazole. (B) Western blotting to validate the purified NTR. (C) Removal kinetics of 0.1 mg l^−1^ CAP by purified NTR with addition of cofactors such as flavin mononucleotide (FMN) and NADPH in individual and combination. Control indicates solution with only CAP. Legends including +FMN, +NADPH, and +FMN + NADPH showed the groups with only addition of CAP and cofactors (without enzymes). Experimental groups with addition of CAP with and without addition of cofactors were marked as +NTR, +NTR + FMN, +NTR + NADPH, and +NTR + FMN + NADPH. (D and E) Enzymatic activities of NTR under different temperature and pH. (F) Michaelis–Menten kinetics for NTR for CAP. (G) Removal kinetics of 0.1 mg l^−1^ CAP in real aquaculture wastewater during 240 min of cultivation. (H) Biolayer interferometry (BLI) sensorgrams depicted the real-time binding of different concentrations of CAP with NTR. (I) Molecular docking showed the binding pockets between CAP and NTR. All experiments were conducted in triplicate (*N* = 3).

To investigate how *Synechocystis* sp. NTR transforms CAP, we conducted heterologous expression and purification of the NTR protein. Sodium dodecyl sulfate–polyacrylamide gel electrophoresis (SDS-PAGE) gel image and Western blotting confirmed successful expression of the target protein (Fig. 4A and B). We then investigated the effect of different cofactors on the removal capacities of NTR toward CAP. As shown in Fig. 4C, we found that the NTR protein is a nicotinamide adenine dinucleotide phosphate (NADPH)-dependent reductase, which has an optimal temperature and pH range of 45 °C and 7.0 to 8.0, respectively (Fig. 4D and E). To further investigate the catalytic efficiency of NTR, we conducted the Michaelis–Menten kinetic analyses (Fig. 4F). The results showed that the Michaelis constant (*K*_m_) of NTR was 104.0 μM, and the maximum reaction rate (*V*_max_) was 3.82 μM min^−1^ (*R*^2^ = 0.95), indicating that *Synechocystis* sp. NTR can directly catalyze CAP.

To evaluate the stability and performance of purified NTR in real aquaculture wastewater scenarios, we investigated the removal kinetics of CAP with addition of NTR. The results showed that NTR treatment removed 52.68% of CAP within 30 min, and complete removal was achieved within 4 h of cultivation (Fig. 4G). The observed degradation rate of CAP in real fish culture wastewater was comparable to those in tris–HCl buffer under optimal pH and temperature, indicating that the NTR enzyme has robustness and potential for practical applications in wastewater treatment. Moreover, compared to the previously observed removal efficiencies, the NTR-induced treatment of CAP can be more efficient (*V*_m_ = 20.44 mg h^−1^). For example, only 85% degradation of 50 mg l^−1^ CAP was achieved at optimal pH of 5 after 24 h of reaction (*V* = 1.77 mg h^−1^) by a bacterial species, *Sphingobium* sp. CAP-1 [46]. Similarly, the isolated *Sphingomonas* sp. CL5.1 achieved complete removal of 120 mg l^−1^ CAP within 48 h (*V* = 2.5 mg h^−1^) [46,47].

To further verify whether NTR can directly bind with CAP, we employed biolayer interferometry. The affinity constant (*K*_d_) obtained was 2.9 nM, indicating a high affinity between NTR and CAP, further highlighting the potential effectiveness of the enzyme in bioremediation or biocatalytic processes (Fig. 4H). To elucidate the binding pockets between NTR and CAP, we performed molecular docking analysis. As shown in Fig. 4I, CAP can interact with the binding residues of TYR8, ARG10, and GLN133 of NTR through hydrogen bonds with a binding energy of −3.94 kcal mol^−1^. This suggests a exothermic and spontaneous binding process between NTR and CAP. Liu et al. [48] also found that Arg^20^ and Trp^71^ in the active site of *Haemophilus influenzae* NTR were the key amino acid residues for CAP binding. Taken together, these characteristics suggest that *Synechocystis* sp. NTR could be a valuable tool in biotechnological applications, particularly in the detoxification and removal of nitroaromatic pollutants. The enzyme’s high affinity and efficient catalytic properties offer distinct advantages, making it a promising candidate for further development and application.

### Identification of transformation products and toxicity assessment

**Fig. 5.**
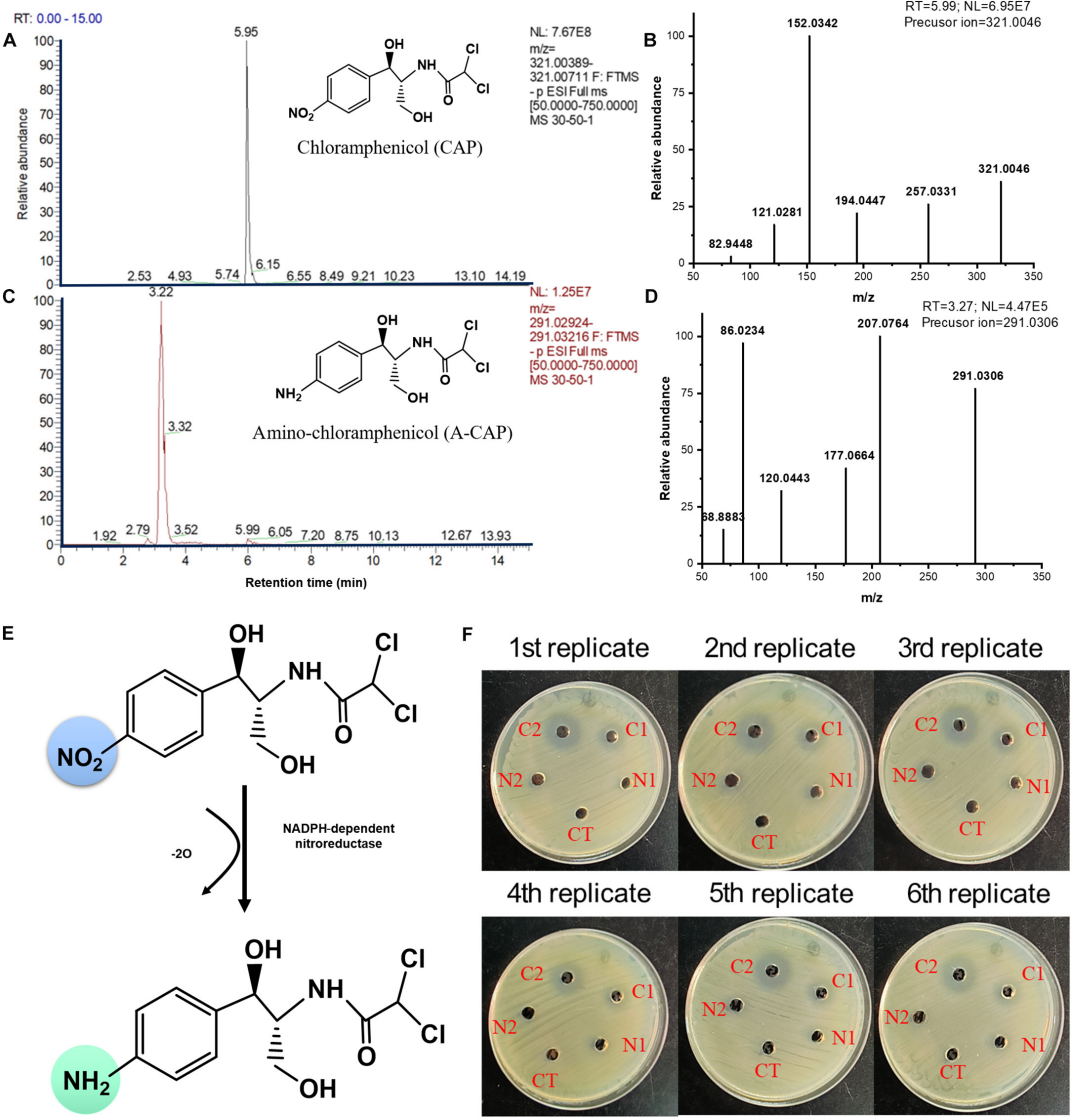
Biotransformation mechanism of CAP by identified NTR. (A and B) Extracted ion chromatography of CAP and its MS^2^ features. (C and D) Extracted ion chromatography of amino-CAP (A-CAP) and its MS^2^ features. (E) Proposed transformation pathway of CAP by *Synechocystis* sp. NTR. (F) Antimicrobial inhibition zone tests to investigate the toxicity of CAP degradation intermediates. CT, control (deactivated enzyme solution); C1, 50 mg l^−1^ CAP; C2, 100 mg l^−1^ CAP; N1, enzyme-treated 50 mg l^−1^ CAP; N2, enzyme-treated 100 mg l^−1^ CAP. The experiment has been conducted 6 times (*N* = 6).

To identify transformed products (TPs) of CAP, we next used high-resolution mass spectrometry to scan the formed TPs in the reaction systems with and without addition of NTR. We found that a unique TP was formed with simultaneously decreasing concentrations of CAP. We extracted the ion chromatographs (EIC) and MS^2^ features of CAP and its TP (Fig. 5A to D). The representative fragmentation ions for qualitative and quantitative analyses of CAP were *m*/*z* 194.0449 and *m*/*z* 152.0342 (Fig. 5B), consistent with the previously reported MS^2^ mass spectrum of CAP [49]. The formed TP (*m*/*z* 291.0306) has representative fragmentation ions of 207.0764, 177.0663, 120.0443, and 86.0233 (Fig. 5D), matching those of amino-CAP produced by *H. influenzae* NTR [50]. These results demonstrated that *Synechocystis* sp. NTR transformed CAP into amino-CAP through the reduction of aromatic nitro groups (Fig. 5E).

We then became curious about the toxicity changes of CAP and its formed TP. We used ecological structure–activity relationship (ECOSAR) toxicity assessments of CAP and amino-CAP (Fig. S8). Calculated EC_50_ values indicated that amino-CAP has lower ecological risks compared to that of CAP on fish, daphnia, and green algae. Additionally, we included toxicity tests such as antimicrobial inhibition zone tests and effect of treated CAP on the growth of *Synechocystis* sp. as the experimental validation of the reduced toxicity of CAP degradation products in the revised manuscript. As shown in Fig. S8, compared to the control, *Synechocystis* sp. grows better in the enzyme-treated initially same concentrations of CAP exposure group compared to the no treated CAP exposure groups (2 and 5 mg l^−1^). Meanwhile, the antibacterial inhibition zone tests also demonstrated that there was no formed inhibition zone by the enzyme-treated CAP compared to the no enzyme-treated CAP groups (Fig. 5F). This suggests that *Synechocystis* sp. NTR significantly reduced the biological toxicity of CAP by reducing its nitro group to an amino group, which demonstrated that NTR can be an environmentally friendly biocatalyst. This further confirmed that *Synechocystis* sp. NTR can be an environmentally friendly biocatalyst or engineered bacteria carrying this gene can be applied in the remediation of CAP-polluted wastewaters.

### Environmental implications

The rapid expansion of aquaculture has intensified environmental challenges, particularly the discharge of antibiotic-laden wastewater, which threatens aquatic ecosystems and contributes to the global spread of antibiotic resistance. This study addresses these challenges by identifying a novel NTR in *Synechocystis* sp. that efficiently degrades CAP through a non-CYP450 pathway. The discovery of this enzyme overturns the conventional assumption that CYP450s dominate antibiotic metabolism in microbial systems, revealing a previously overlooked biocatalytic mechanism. By heterologously expressing NTR in *E. coli*, we demonstrated its capacity to remove >90% of CAP within 2 h, far surpassing the efficiency of natural bacterial strains and conventional sludge-based treatments. This enzymatic process converts CAP into less toxic amino-CAP, significantly reducing ecological risks to aquatic organisms, as validated by toxicity assays and molecular docking.

Furthermore, canonical correspondence (CCA) and redundancy analysis (RDA) correlation analyses revealed that the abundance and transcriptional activity of NTR genes across microbial taxa worldwide were strongly correlated with distinct environmental gradients, reflecting ecological partitioning and niche specialization (Fig. S6). The CCA model based on NTR gene distribution abundance explained 14.9% (CCA1) and 8.6% (CCA2) of the environmental variances in microbial community composition, with phosphate (importance value = 0.42), oxygen (0.41), salinity (0.28), nitrate/nitrite (0.25), and temperature (0.24) emerging as key drivers of NTR-harboring microbial distribution. NTR gene abundance distributed in Bacteroidetes, Gammaproteobacteria, Betaproteobacteria, and other Proteobacteria exhibited strong positive correlations with oxygen, chlorophyll a, nitrate/nitrite, and phosphate levels. These bacterial phyla have been proved with dominance in nutrient-rich, oxygenated environments such as coastal surface waters [51,52]. Conversely, NTR gene abundance distributed in Chloroflexi, Alphaproteobacteria, Verrucomicrobia, and Cyanobacteria showed negative correlations with oxygen, nitrate/nitrite, and phosphate, but positive associations with salinity, temperature, and iron concentrations. Previous research showed that under varying redox and nutrient-rich conditions, marine microbial communities exhibit an enrichment in the abundance and expression of genes encoding reductase and oxidase [53–56]. NTR gene abundance distributed in Firmicutes and Planctomycetes displayed broad positive correlations with most environmental factors except oxygen and chlorophyll a. This indicated that there might be an antagonistic relationship between oxygen and chlorophyll producing phyla and Firmicutes and Planctomycetes. RDA analysis of NTR transcript abundance across taxa explained 14.6% (RDA1) and 4.3% (RDA2) of the total environmental variance, with temperature (0.38) and oxygen (0.35) exerting the strongest influence on transcriptional activity. NTR transcripts in Bacteroidetes correlated positively with oxygen, chlorophyll a, nitrate/nitrite, phosphate, and iron, factors typical of eutrophic, oxygenated surface waters [52]. In contrast, NTR transcripts in Gammaproteobacteria and Planctomycetes showed positive associations with salinity, temperature, coastal proximity, iron, nitrate/nitrite, and phosphate, but negative links to oxygen and chlorophyll a. The observed niche-specific transcriptional patterns underscore the adaptive strategies of microbial communities to environmental heterogeneity [54], with NTR-mediated processes playing critical roles in biogeochemical cycling and antibiotic detoxification across marine ecosystems.

By bridging the gap between enzymatic discovery and practical application, this work provides a sustainable biotechnology to mitigate antibiotic pollution. Engineered bacteria expressing *Synechocystis* NTR could be deployed in wastewater treatment systems to rapidly detoxify CAP, curbing the selection of resistant genes and protecting aquatic biodiversity. The integration of such targeted biocatalysts into circular aquaculture practices, where microalgal biomass from treated wastewater is repurposed as feed or biofertilizer [57], offers a dual benefit of reducing environmental footprints while enhancing resource efficiency. These findings advance the development of precision bioremediation tools, aligning aquaculture growth with planetary health imperatives in the era of escalating antimicrobial resistance [58]. While engineered bacteria offer exciting opportunities for advancing sustainable aquaculture, their application faces significant challenges. These include environmental and ecological risks, such as horizontal gene transfer; technical limitations, such as ensuring long-term stability and efficacy; and practical hurdles in monitoring and control, including the need for real-time tracking systems.. Further studies should be conducted to balance innovation with safety and environmental stewardship.

## Materials and Methods

### Chemicals

CAP (CAS 56-75-7, purity >98%) was purchased from Sigma-Aldrich (Shanghai, China). High-performance liquid chromatography (HPLC)-grade water, acetonitrile, methanol, and formic acid were obtained from Merck (Darmstadt, USA). The other chemicals were obtained from Sinopharm (Beijing, China).

### *Synechocystis* sp. tolerance and degradation capacity assessment

*Synechocystis* sp. FACHB 898 was purchased from the Institute of Aquatic Biology of the Chinese Academy of Sciences (CAS; Wuhan, China). Effects and removal kinetics of different concentrations (0.1, 1, 1.25, 1.5, 2, and 5 mg l^−1^) of CAP on *Synechocystis* sp. were investigated in sterilized BG11 medium in a shaking incubator (10,000 lux, a light ratio of 16 h:8 h, and 27 °C). The initial inoculum size was set as 1.0 × 10^6^ cells ml^−1^. Unless specially mentioned, all experiments have been conducted in the abovementioned conditions in triplicate (*N* = 3). Cell number was counted to investigate the relative growth inhibition caused by different concentrations of CAP according to our previous protocol [59]. We also used a dose–response model named inhibitor versus response–variable slope (4 parameters) to assess the inhibitory data, and details can be found in Text S1.

Concentration changes of added CAP in the medium were determined using an HPLC (Alliance e2695 system, Waters, USA) coupled with an ultraviolet detector (2489). The elution of CAP was achieved by a Zorbax C18 column (250 × 4.6 mm, 5 μm) by running a mobile phase (acetonitrile:water:formic acid = 4:6:0.01). The detection wavelength and flow rate were 278 nm and 1 ml min^−1^, respectively. Removal kinetics were analyzed using a first-order model [59,60]. Mass balance of CAP removals was calculated to quantify the removed amounts by abiotic factors, bioadsorption, bioaccumulation, and biodegradation according to our reported method [59,60]. Details were shown in Text S2.

### Assessment of CYP450s’ role

To investigate whether CYP450s determine the removal efficiency of CAP in *Synechocystis* sp. FACHB 898 culture, we used ABT to inhibit the enzymatic activities of CYP450s [35,36]. In brief, the effect of different concentrations (5, 10, 50, and 100 μM) of ABT on the removal of 0.1 mg l^−1^ CAP by *Synechocystis* sp. was monitored during 14 d of cultivation.

### Expression analysis of a gene encoding cyanobacterial NTR

To explore the expression levels of a cyanobacterial NTR (located in the region from 1,470,319 to 1,470,924 in the chromosome of *Synechocystis* sp. (CP073017.1; https://www.ncbi.nlm.nih.gov/nuccore/CP073017.1?from=1470319&to=1470924), total RNA of *Synechocystis* sp. FACHB 898 was extracted using an RNA extraction reagent (Vazyme, R401). Primers for the NTR gene were designed by Primer Premier 5.0. Primers’ amplification specificity and amplification efficiency were achieved by the agarose gel electrophoresis. Real-time quantitative polymerase chain reaction (RT-qPCR) was performed using a 2-step method with the Taq Pro Universal SYBR qPCR Master Mix (Vazyme, Q712-02) according to the manufacturer’s instructions.

### Removal of CAP by a bacterial species carrying NTR

To verify whether *Synechocystis* sp. NTR plays an essential role in the removal of CAP, we cloned its encoding gene in *E. coli*. Then, we investigated the removal kinetics of 0.1 mg l^−1^ CAP by the wild and engineered *E. coli*. In brief, the sequence encoding *Synechocystis* sp. NTR was PCR-cloned into the pET28a-sumo vector with the N terminus followed by 6-His tag using Bam HI restriction site to generate pNTR. The RNA sequencing was confirmed by Sangon Biotech Co. Ltd. (Shanghai, China). The right recombinant vector was next transformed into chemically competent *E. coli* BL21 (DE3) by heat shock at 42 °C followed by recovery for 3 h at 37 °C in Luria Broth (LB) medium. *E. coli* containing vector-expressed NTR were inoculated into LB medium with 50 μg ml^−1^ kanamycin at 37 °C until the culture reached an OD_600_ (optical density at 600 nm) of 0.6 to 0.8. Then, the cultivated *E. coli* containing NTR with and without induction by 0.1 mM IPTG was used to remove 0.1, 1, 5, and 10 mg l^−1^ CAP in a mineral medium with an inoculum size of 1.0% for 6 h at 37 °C. Samples were withdrawn at times of 0.5, 1, 2, 4, and 6 h to determine the residual CAP amounts in the solutions. All experiments were conducted in triplicate (*N* = 3).

### Distribution of NTR through metagenomics/metatranscriptomics

The geographic distribution of the NTR gene was determined by querying its protein sequences (GenBank: QWO81845.1) against the prokaryote-enriched Oceans Microbiome Reference Gene Catalog Dataset [37], with a threshold *E* value ≤ 1 × 10^−10^ via the Ocean Gene Atlas (https://tara-oceans.mio.osupytheas.fr/ocean-gene-atlas/) [61,62]. Briefly, the Tara Oceans metagenomes (OM-RGC_v2_metaG) and metatranscriptomes (OM-RGC_v2_metaT) were searched using a blastp method for extracting the sequences and abundances. The percent of mapped read default normalization was applied for estimating the unigene abundance. The normalization divides the homologs’ read coverage by the total number of reads for the sample. The outputs included alignment results, homolog sequences, FASTA files, normalized abundances of the homologs, and environmental data, which were listed in Tables S4 and S5. For the biogeographic distribution pie chart, detailed sample information, taxonomy, and homolog abundance data from different sample depths were provided in Tables S6 to S11. To analyze the correlations between NTR gene abundance and transcripts across different taxonomic phyla and environmental parameters, we applied CCA/RDA models. Details regarding the RDA and CCA analyses can be found in Text S3.

### Protein heterologous expression and functional characterization

To further characterize the function of cyanobacterial NTR, protein expression of *E. coli* carrying NTR gene was induced by the addition of 0.1 mM IPTG, which was grown for 18 h at 19 °C. Cells were collected by centrifugation at 6,000 rpm for 6 min at 4 °C and resuspended in phosphate-buffered saline (PBS) buffer (137 mM NaCl, 2.68 mM KCl, 10.14 mM Na_2_HPO_4_, 1.76 mM KH_2_PO_4_, pH 8.0). The cell suspension was then sonicated (400 W, with a pulsing protocol of 2 s on and 3 s off for 15 min) and centrifuged at 11,000 rpm for 20 min at 4 °C. The whole-cell lysates, supernatants, and inclusion bodies were analyzed by SDS-PAGE against the target protein. The supernatants containing NTR were added into a Ni-NTA column equilibrated in the 30-ml TBS buffer (50 mM tris, 100 mM NaCl). The protein was then eluted using different concentrations of imidazole (0 to 110 mM). The eluent solutions were subsequently analyzed by SDS-PAGE. Solutions with NTR were concentrated by ultrafiltration tube and stored at −80 °C. Western blot verification and functional characterization of *Synechocystis* sp. NTR can be found in Texts S4 and S5.

To test the stability and performance of purified NTR in real aquaculture wastewater, we sampled the zebrafish culture wastewater (pH 7.21, chemical oxygen demand 24 mg l^−1^, total nitrogen 4.19 mg l^−1^, total phosphorus 0.01 mg l^−1^, and ammonium 0.36 mg l^−1^), which was further used as the enzyme reaction solution. We then added 0.1 mg l^−1^ CAP, 1 μM NTR, and 1 mM cofactor NADPH to the wastewater and incubated the mixture at 28 °C for 4 h. Samples have been withdrawn at times of 0, 15, 30, 60, 120, 180, and 240 min to determine the residual CAP amount in the solution. This experiment has been conducted 4 times (*N* = 4).

### Binding mode assessment between NTR and CAP

Biolayer interferometry (BLI) measurement was performed on the Octet Red96 system (Pall Fortebio, USA) to investigate the binding affinity between NTR and CAP. The concentrations of NTR and NADPH were 1 μM and 1 mM, respectively, while the concentration gradients of CAP were set as 7.7, 15.5, 30.9, and 61.9 mM. All binding data were collected at 25 °C. The assays included 4 steps: (a) baseline acquisition for 60 s, (b) biotinylated NTR loading onto sensor for 300 s, (c) association with CAP for 300 s, and (d) dissociation of CAP. Baseline and dissociation steps were carried out in 0.002% PBST buffer (PBS with 0.1% Tween 20). Molecular docking was used to predict the binding pockets between CAP and NTR, and details can be found in Text S6.

### Identification of biotransformation product by NTR

Samples containing enzyme and/or CAP were collected to determine the biotransformation product of CAP to unravel the degradation mechanism of NTR during 60 min of cultivation. To investigate the formed transformation product, we set 3 groups including only NTR, NTR with 0.1 mg l^−1^ CAP, and only CAP. All the samples have been collected at 0, 30, and 60 min and monitored by a Q Exactive Orbitrap Mass Spectrometers (Thermo Fisher Scientific, USA) with Thermo Hypersil gold column (2.1 mm × 100 mm, 1.9 μm). Toxicity changes of enzyme-catalyzed CAP were also investigated using analyses such as ECOSAR toxicity assessments, antimicrobial inhibition zone tests, and effect of treated CAP on the growth of *Synechocystis* sp. Details can be found in Text S7.

## Data Availability

The data that support the findings of this study are available from the corresponding author, [Xiong J.Q. xiongjiuqiang@ouc.edu.cn], upon reasonable request.

## Supplementary Materials

Supplementary 1 Texts S1 to S7 Figs. S1 to S8 Tables S1 to S11
