# Supplementary material for: A Globally Distributed Cyanobacterial Nitroreductase Capable of Conferring Biodegradation of Chloramphenicol
Source: Research (Wash D C). 2025 May 8;8:0692. doi: 10.34133/research.0692 (PMC12063702; doi:10.34133/research.0692)
Supplement: Supplementary 1 — Texts S1 to S7 Figs. S1 to S8 Tables S1 to S11 [file research.0692.f1.zip › supporting information.docx]

***Supporting Information for***

**A globally distributed cyanobacterial nitroreductase capable of conferring biodegradation of chloramphenicol**

Qiu-Lian Zhong, Jiu-Qiang Xiong*

College of Marine Life Sciences, Ocean University of China, Yushan Campus, Yushan Road 5, Qingdao, Shandong, China.

*Correspondence: Xiong J.Q. ([xiongjiuqiang@ouc.edu.cn](mailto:xiongjiuqiang@ouc.edu.cn))

There are 24 pages in SI, including 7 texts, 8 figures, 11 tables, and 1 reference.

**Supplementary text 1.** **Dose-Response Model Analyses**

Cell numbers were measured by a hemocytometer under a microscope (Nikon H600L, Tokyo, Japan). Relative growth inhibition was calculated by comparing the difference in the cell numbers between treatment group and control. Then, we used an inhibitor vs. response-variable slope (four parameters) model to assess the inhibitory data. These four parameters include Top, Bottom, Hill slope, and the EC_50_ (or IC_50_). The Top and Bottom parameters describe the values at which the curve reaches a plateau - coming infinitely close, but never quite reaching these values. The Hill slope describes the slope of the sigmoidal curve between these two plateaus. The EC_50_ (or IC_50_) refer to a concentration of agonist (or antagonist) required to increase (or reduce) the measured response to half- or 50%- of its maximal value.

$$Y=Bottom+\frac{Top-Bottom}{1+\frac{EC50/IC50}{X}^^{HillSlope}}$$

Where X is the real concentrations of chloramphenicol at time t; Y is the relative inhibition percentage of the microalgal cell numbers at time t.

**Supplementary text 2.** **Removal Kinetics and Mass Balance Analyses of Chloramphenicol Removal.**

The removal kinetics of chloramphenicol (CAP) by *Synechocystis* sp. FACHB 898 were analyzed using a first-order reaction model as follows:

$$LnC_{t}=-kt+{LnC}_{0}$$

where C_0_ is the initial concentration of CAP at time zero, C_t_ is the concentration of CAP at time t, k is the removal rate constant (d^−1^), and t is the removal period in days.

Mass balance of the removal of CAP was analyzed to show the removed amounts caused by abiotic factors, bioadsorption, bioaccumulation, and biodegradation. To measure the residual amount in the culture medium, the algal suspensions were centrifuged at 4500 rpm for 10 min, and the supernatant recovered after centrifugation was used to determine the residual amphenicols in the culture medium. The difference between initial concentration of added compound and residual amount in solution has been defined as total removal. Removal induced by abiotic factors has been monitored according to the concentration changes of the CAP in the control group (only existing of antibiotics) during experimental duration under same conditions. The harvested cell pellets were washed and suspended with distilled water (10 mL) and then centrifuged. CAP in the supernatant were further concentrated using a solid-phase extraction method, which was used to determine the amount of CAP adsorbed onto the cell surface. The harvested cell pellets were mixed with 3 mL of dichloromethane : methanol (1:2 v/v) for sonication (40 kHz, 2.2 Kw, 1 h), which was centrifuged at 4500 rpm for 10 min. The supernatant retrieved after centrifugation was used to determine the quantity of CAP that was accumulated within the microalgal cells. The biodegradation percentage (*P*_b_) of CAP induced by the microalgal species was calculated according to the following equation;

$$P_{b}\left( \% \right)=(A_{t}-A_{r}-A_{d}-A_{a}-A_{c})\frac{100}{A_{t}}$$

where, *A_t_* is the initial concentration of CAP added to the medium, *A_r_* is the residual amount of CAP in the medium, *A_d_* is the amount of CAP adsorbed on the microalgal cell surface, *A_a_* is the amount of CAP removed by abiotic processes and *A_c_* is the amount CAP accumulated in the microalgal cells.

**Supplementary text 3.** To analyze the correlations between NTR gene abundance and transcripts across different taxonomic phyla and environmental parameters, we applied CCA/RDA models. In a brief, data from the Tara Oceans metagenomes (OM-RGC_v2_metaG) and metatranscriptomes (OM-RGC_v2_metaT) databases were first classified and organized into abundance matrices, corresponding environmental factors, and grouping files, which were then imported into the CCA/RDA framework. The species abundance table data were subjected to Detrended Correspondence Analysis (DCA) to assess the gradient lengths of ordination axes. Based on the axis lengths derived from DCA, the appropriate model was selected: if the gradient length exceeded 3.0 units, the CCA model was chosen to account for unimodal species-environment relationships typical of long ecological gradients, whereas the RDA model (assuming linear responses) was applied when the gradient length was below 3.0. This approach ensured optimal alignment between the statistical model and the underlying ecological gradients in the dataset. Redundancy Analysis (RDA) and Canonical Correspondence Analysis (CCA) are constrained ordination techniques derived from correspondence analysis (CA), designed to explicitly model relationships between biological communities (e.g., microbial taxa) and environmental variables. RDA assumes linear species-environment relationships. It begins by fitting a multivariate linear regression model where the species abundance matrix (Y) is predicted by the environmental matrix (X). The fitted values (Ŷ = XB) represent the portion of species variation explained by environmental factors. These fitted values are then subjected to principal component analysis (PCA) after double-focused standardization (adjusting row and column weights), generating constrained axes that capture gradients of environmental influence. Residual variation unexplained by the model is further explored through PCA. RDA is particularly effective for short environmental gradients where species exhibit monotonic responses, such as linear effects of pH or nutrient levels on microbial composition.

In contrast, CCA operates under the unimodal response hypothesis, where species distributions are assumed to have optimal ranges along environmental gradients. CCA addresses compositional data with high zero-inflation (e.g., sparse microbial datasets) by incorporating chi-square distances through weighted regression. The species matrix is weighted by row (sample totals) and column (species frequencies) to balance the influence of rare and abundant taxa. The weighted matrix is regressed onto environmental variables, and the resulting fitted values are decomposed via correspondence analysis to produce canonical axes. These axes maximize the covariance between species distributions and environmental predictors while preserving the chi-square metric, making CCA suitable for long gradients or datasets with strong niche-based species responses, such as biogeographic studies of microbiota across diverse habitats. Both methods are termed direct gradient analyses due to their iterative integration of regression and ordination, enabling explicit modeling of environmental effects on community structure.

Detrended correspondence analysis (DCA) is an ordination technique developed to address the "arch effect," a common artifact in traditional correspondence analysis (CA) or canonical correspondence analysis (CCA) that arises when analyzing ecological datasets with long environmental gradients. The arch effect manifests as a curved or horseshoe-shaped distortion in ordination plots, where the second axis becomes a quadratic function of the first axis, obscuring true ecological gradients. DCA mitigates this distortion through detrending and rescaling procedures, aiming to linearize species-environment relationships for clearer interpretation. The method begins with a standard CA decomposition of the species abundance matrix to derive initial ordination axes. To eliminate the arch effect, DCA divides the first axis into segments of equal width and performs local adjustments within each segment. Specifically, the second-axis coordinates within each segment are centered (e.g., by subtracting segment-specific means or fitting low-order polynomials), effectively removing the quadratic dependency on the first axis. This detrending process is iterated across higher-order axes until nonlinear distortions are minimized. Subsequently, the ordination axes are rescaled to standardize gradient lengths based on species turnover rates, ensuring that each unit along the axes represents ecologically interpretable changes in community composition (e.g., a full species turnover typically spans approximately 4 rescaled units).

**Supplementary text 4. Western Blotting Verification.**

The purified proteins were denatured by heating at 95°C for 7 minutes in a loading buffer and separated on an SDS-PAGE gel. The separated proteins were transferred to a Polyvinylidene Fluoride (PVDF) membrane using a wet transfer system (100 V for 90 min). Then the membrane was blocked with 5% non-fat dried milk in PBST buffer (137 mM NaCl, 2.68 mM KCl, 10.14 mM Na_2_HPO_4_, 1.76 mM KH_2_PO_4_, 1mL Tween-20 for 1 L, pH 7.4) for 3 hs to prevent non-specific binding. After that, the membrane was washed using the PBST buffer for 15 min. The membrane was incubated overnight at 4°C with the HRP-conjugated Anti-His mouse monoclonal antibody and an HRP-conjugated Goat Anti-Mouse IgG (Sangon Biotech, China) for 1 h at room temperature. Subsequently, the membrane was treated with enhanced chemiluminescence (ECL) substrate (Biosharp, BL520A, China) to generate a chemiluminescent signal, which was detected using an imaging system.

**Supplementary text 5. Functional Characterization of *Synechocystis* sp. Nitroreductase.**

To investigate the effect of cofactors on the removal of CAP by purified NTR, a 10 mL enzyme reaction system was set up with final concentrations of 3 µM CAP, 10 µM FMN, 1 mM NADPH, and 1 µM enzyme (NTR). The reaction was conducted in a water bath at 37°C for 180 min. Samples were withdrawn at a regular time interval to quantify residual CAP in the solution. To investigate the optimal pH for *Synechocystis* sp. NTR activity, a 5 mL enzyme reaction system was set up with final concentrations of 3 µM CAP, 1 mM NADPH, and 1 µM enzyme. The buffer pH was varied from 3.0 to 9.0, and the reaction was incubated at 37°C for 30 min. To investigate the optimal temperature of NTR, the incubation temperatures varied from 25 to 75°C in the optimal pH for 30 min. To determine the Michaelis-Menten kinetics of CAP, the removal of eight different concentrations of CAP (0.3, 3, 15, 30, 150, 300, 600, and 900 µM) by NTR was investigated at the optimal pH and temperature for 30 min.

**Supplementary text 6. Molecular Docking.**

Three-dimensional structure of the *Synechocystis* sp. NTR, CAP, and NADPH were obtained from UniProt (Entry: Q55233) and PubChem (CAS 56-75-7, and CAS 53-57-6), respectively. The downloaded sdf formats of CAP and NADPH were converted to pdb format using the Open Babel software. Given that the action of NTR requires the cofactor NADPH, NADPH was first docked to NTR using AutoDock 4.0. Subsequently, CAP was docked to the binary complex of NTR and NADPH. The detailed steps were as follows. The receptor (CAP and NADPH) structures were initially cleaned by removing water molecules, and then hydrogen atoms and charges were added. The protein structure was similarly cleaned by adding hydrogen atoms, charges, and root. Based on the size and position of the modified NADPH and NTR, we optimized the configuration of NADPH and determined the center grid box. AutoGrid was then performed to prepare the grid maps, followed by launching AutoDock to perform the docking simulation. After that, the top-ranked poses are analyzed based on the estimated binding free energy. The conformation with the lowest binding free energy was selected as the most likely binding sites. CAP was then docked to the binary complex of NTR and NADPH following the same docking procedure. The final conformation with the lowest binding free energy was selected. The structures were visualized using PyMOL software.

In details, acquisition of Protein and Ligand Structures: The three-dimensional structure of the *Synechocystis* sp. NTR was obtained from UniProt (Entry: Q55233), while the structures of CAP (CAS 56-75-7) and NADPH (CAS 53-57-6) were retrieved from PubChem. The molecular structures of CAP and NADPH were initially downloaded in SDF format. Conversion of Ligand Formats: The SDF files for CAP and NADPH were converted into PDB format using Open Babel software. This conversion was required to make the ligands compatible with AutoDock 4.0 for molecular docking simulations. Docking of NADPH to NTR: As the action of NTR requires the cofactor NADPH, NADPH was first docked to NTR using AutoDock 4.0. Both the NTR protein structure and NADPH cofactor were prepared for docking by cleaning the structures. This step included the removal of water molecules, addition of hydrogen atoms, and assignment of partial charges. Similarly, the NTR protein structure was cleaned by adding hydrogen atoms, partial charges, and a root. Grid Generation and Docking Preparation: The configuration of NADPH was optimized based on the size and position of the modified NADPH and NTR. The center of the grid box was then determined, and AutoGrid was used to prepare the grid maps required for docking simulations. These grid maps define the search space for docking simulations. Docking Simulation: Docking simulations were performed using AutoDock 4.0. First, the binary complex of NTR and NADPH was prepared, and the docking process was executed to simulate the binding of NADPH to the protein. The top-ranked docking poses were selected based on their estimated binding free energy, which is calculated during the docking process. Selection of the Best Binding Conformation: The conformation with the lowest binding free energy was selected as the most likely binding site. This conformation represents the most energetically favorable interaction between NADPH and NTR. Docking of CAP to the NTR-NADPH Complex: Following the same docking procedure, CAP was docked to the binary complex of NTR and NADPH. The receptor complex, consisting of both NTR and NADPH, was prepared, and CAP was introduced into the docking simulation. After performing the docking simulation, the final conformation with the lowest binding free energy was selected as the most likely binding pose for CAP. Visualization of the Structures: The structures of the binary complex and the final docking poses were visualized using PyMOL software. This software was used to analyze the binding interactions and visualize the docking results, helping to identify key interactions between the molecules.

**Supplementary text 7. Identification of Transformation Products.**

Solvents including 2mM ammonia water (A), and methanol (B) were used as the mobiles phase. The injection volume was 5 µL with a flow rate of 0.35 mL min^-1^. Gradient elution was set as follows: 0~1 min, 0-5 % B; 1~11 min, 5-100 % B; and 11~15 min, 100-5 % B. Data were acquired at negative mode by full MS1 scan (50–1050) followed by data-independent acquisition (DIA). The other main parameters were set as follows: (N) CE/stepped nce, 20 and 40; nitrogen gas flow rate, 600 L h^-1^; nebulizer temperature, 350℃; ion source temperature, 150℃; and cone gas hole flow rate, 30 L h^-1^. For the toxicity tests, purified NTR (2 µM) and coenzyme NADPH (1 mM) were used to degrade 80 and 200 mg/L CAP in a water bath at 45°C. After allowing the reaction to proceed fully for 1 hour, the resulting degradation mixtures were added to 250 mL conical flasks containing 100 mL of BG11 medium to achieve an expected initial concentration of 2 and 5 mg/L CAP. Control groups were set up with the same volume of buffer containing inactivated NTR, 2, and 5 mg/L of CAP. Group with only inoculum of microalgal cells was also set up. The initial inoculation concentration of *Synechocystis* sp. FACHB 898 in 100 mL of medium was 1.0×10^6 cells/mL. Each group was set up in triplicates (N=3), and the algal cell density was monitored every 24 hours using a fluorescence microscope. Additionally, an inhibition zone experiment was conducted to evaluate the toxicity of the degradation products to *Escherichia coli*. Similarly, purified NTR (2 µM) and cofactor NADPH (1 mM) were added into the 50 and 100 mg/L of CAP solution, which were inoculated in a water bath at 45°C. After allowing the reaction to proceed fully for 1 hour, 50 µL of the degradation mixture was added to the wells of a solid agar plate pre-inoculated with *E. coli*. Control groups included 50 mg/L and 100 mg/L CAP solutions (no enzyme treated) and buffer containing inactivated NTR. After adding the solutions, the plates were incubated at 37°C for 16 hours, and the inhibition zones were observed and recorded. All experiments have been conducted in six times (*N*=6).

**Supplementary Table 1.** Primers for amplifying nitroreductase gene bp606.

| Primer Name | Sequence(5'-3') | Restriction enzyme |
| --- | --- | --- |
| NTR-S | AGAGAACAGATTGGTGGATCCATGGACACCTTTGACGCTATTTACC | BamH Ⅰ |
| NTR-AS | ACGGAGCTCGAATTCGGATCCTTAGGCAAAGGAGTTTTCCCAGAC |  |

**Supplementary Table 2.** Fitting parameters of chloramphenicol toxicity data at day 2, 4, 6, 8, 10, 12 and 14 using log (inhibitor) and response-variable slope (four parameters) models.

| Items | Days | | | | | | |
| --- | --- | --- | --- | --- | --- | --- | --- |
|  | 2 | 4 | 6 | 8 | 10 | 12 | 14 |
| Best fitted value | | | | | | | |
| Bottom | -0.7272 | -7.130 | 2.333 | -11.34 | -1.805 | -1.709 | 1.874 |
| Top | 91.62 | 98.35 | 95.57 | 90.50 | 82.56 | 86.01 | 86.84 |
| EC50 | 1.149 | 1.096 | 1.522 | 1.487 | 1.581 | 1.816 | 2.000 |
| HillSlope | 1.920 | 3.276 | 5.605 | 4.456 | 7.993 | 6.255 | 8.433 |
| Log EC50 | 0.06031 | 0.03974 | 0.1824 | 0.1722 | 0.1990 | 0.2591 | 0.3009 |
| Span | 92.35 | 105.5 | 93.24 | 101.8 | 84.37 | 87.72 | 84.96 |
| 95% confidence interval | | | | | | | |
| Bottom | -231.1 - 9.101 | -11.32 --2.941 | -11.77 - 14.80 | -23.42 - 0.2452 | -8.849 - 4.630 | -9.056 - 4.904 | -6.159 - 8.273 |
| Top | nc | 94.01 - 103.0 | 81.37 - 110.6 | 78.31 - 103.8 | 73.64 - 91.66 | 76.89 - 95.23 | 76.85 - 96.84 |
| EC50 | nc | 1.042 - 1.150 | 1.349 - 1.733 | 1.327 - 1.677 | 1.484 - 1.699 | 1.680 - 1.963 | 1.862 - 2.196 |
| HillSlope | nc | 2.698 - 3.937 | 3.134 - 15.29 | 2.851 - 7.243 | 5.463 - 17.57 | 4.226 - 9.359 | nc |
| Log EC50 | nc | 0.01782 -0.06086 | 0.1299 - 0.2388 | 0.1227 - 0.2247 | 0.1715 - 0.2302 | 0.2252 - 0.2929 | nc |
| Goodness of fit | | | | | | | |
| R^2^ | 0.9425 | 0.9926 | 0.9174 | 0.9428 | 0.9658 | 0.9623 | 0.9519 |
| Sy.x | 7.501 | 3.376 | 11.69 | 9.915 | 7.165 | 7.271 | 8.045 |

Note: nc indicated not calculated.

**Supplementary Table 3.** Parameters of removal kinetics analyses of chloramphenicol.

| Concentrations | Removal constant (k, d^-1^) | Half-lives (T_1/2_, d) | R^2^ |
| --- | --- | --- | --- |
| 0.1 | 0.2765 | 2.51 | 0.97 |
| 1 | 0.1634 | 4.24 | 0.97 |
| 1.25 | 0.1360 | 5.10 | 0.90 |
| 1.5 | 0.1055 | 6.57 | 0.91 |
| 2 | 0.0593 | 11.69 | 0.94 |
| 5 | 0.0633 | 10.95 | 0.91 |

**Supplementary Figure 1.** Expression and purification of *Synechocystis* sp. nitroreductase from *E. coli.* (A) Sodium dodecyl sulfate-polyacrylamide gel electrophoresis of *E. coli.* containing empty vector (1, 2, 3, 4) or vector-expressed NTR (5, 6, 7, 8) after sonication and centrifugation. (1)&(5)：*E. coli.* samples before IPTG induction; (2)&(6): *E. coli.* samples after IPTG induction; (3)&(7): Supernatant after ultrasonic crushing and centrifugation of the bacterial solution; (4)&(8): Inclusion bodies after ultrasonic crushing and centrifugation of the bacterial solution. (B) Western blot of *E. coli* containing vector-expressed NTR (5, 6, 7, 8). M indicates Marker.

**Supplementary Figure 2.** Growth patterns of used *Synechocystis* sp. in presence of 0 (CT) and 0.1 mg L^-1^ (CAP) chloramphenicol with different concentrations of cytochrome P450 enzyme inhibitor 1-aminobenzotriazole. All experiments have been conducted in triplicates (*N*=3).

**Supplementary Figure 3.** Removal kinetics of different concentrations of CAP by *Escherichia coli* carrying nitroreductase gene ((+) vector), and *Synechocystis* nitroreductase gene free (empty vector) during 6 hours of cultivation. The used *Escherichia coli* was with the induction by the isopropyl β-D-1-thiogalactopyranoside (IPTG). All experiments have been conducted in triplicates.


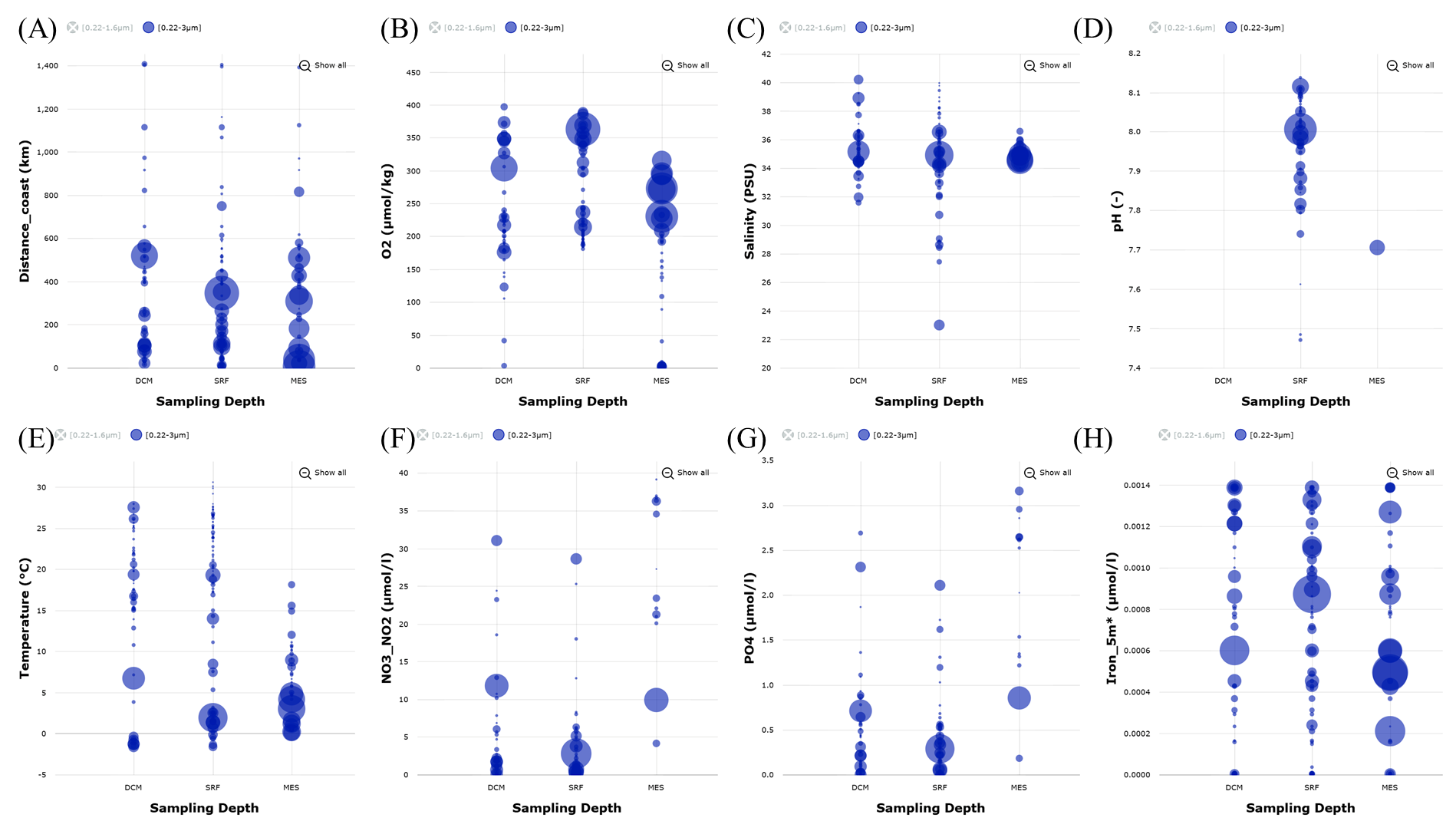


**Supplementary Figure 4.** The influence of environmental factors on the geological distribution of the nitroreductase (NTR) gene homologs at a global scale. (A) Distance coast (km); (B) O_2_ (µmol/kg); (C) Salinity (PSU); (D) pH; (E) Temperature (°C); (F) NO_3_^-^-NO_2_^-^ (µmol/L); (G) PO_4_^3-^ (µmol/L); (H) Iron_5m (µmol/L).


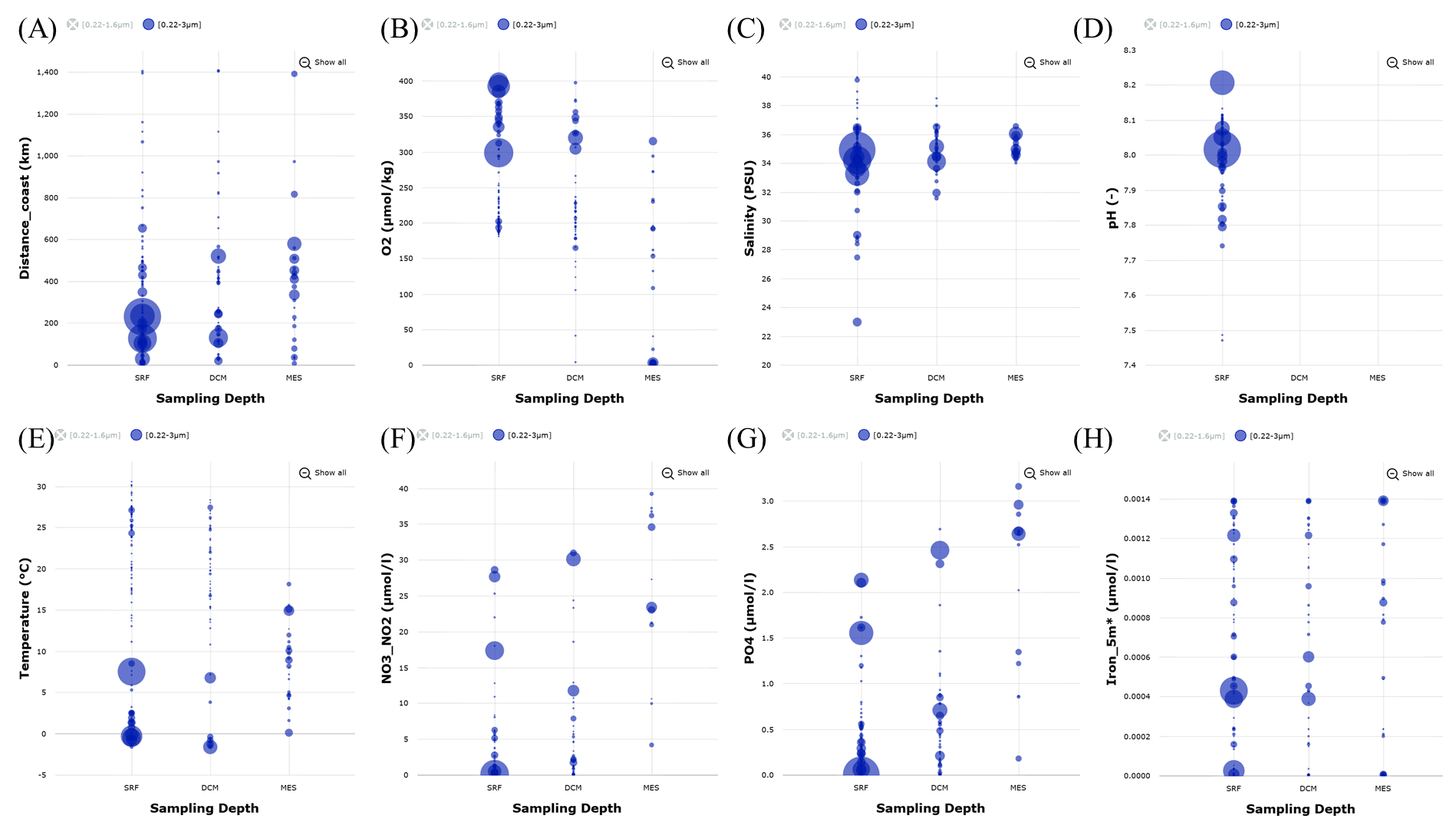


**Supplementary Figure 5.** The influence of environmental factors on the geological transcript abundance of the nitroreductase (NTR) gene homologs at a global scale. (A) Distance coast (km); (B) O_2_ (µmol/kg); (C) Salinity (PSU); (D) pH; (E) Temperature (°C); (F) NO_3_^-^-NO_2_^-^ (µmol/L); (G) PO_4_^3-^ (µmol/L); (H) Iron_5m (µmol/L).

**Supplementary Figure 6.** Correlations between NTR gene distribution abundance (A) and transcripts (B) across different taxonomic phyla in different microbial taxa in surface (SRF), deep chlorophyll maximum layer (DCM), and the mesopelagic layer (MES) and environmental parameters. Environmental factors include distance coast, oxygen levels, chlorophyll A amount, salinity, temperature, nitrate, nitrite, total phosphase, and iron concentrations. The correlation analyses have been performed using canonical correspondence /redundancy analysis (CCA/RDA) models. Details of the environmental factors can be found in Table S4-S11.


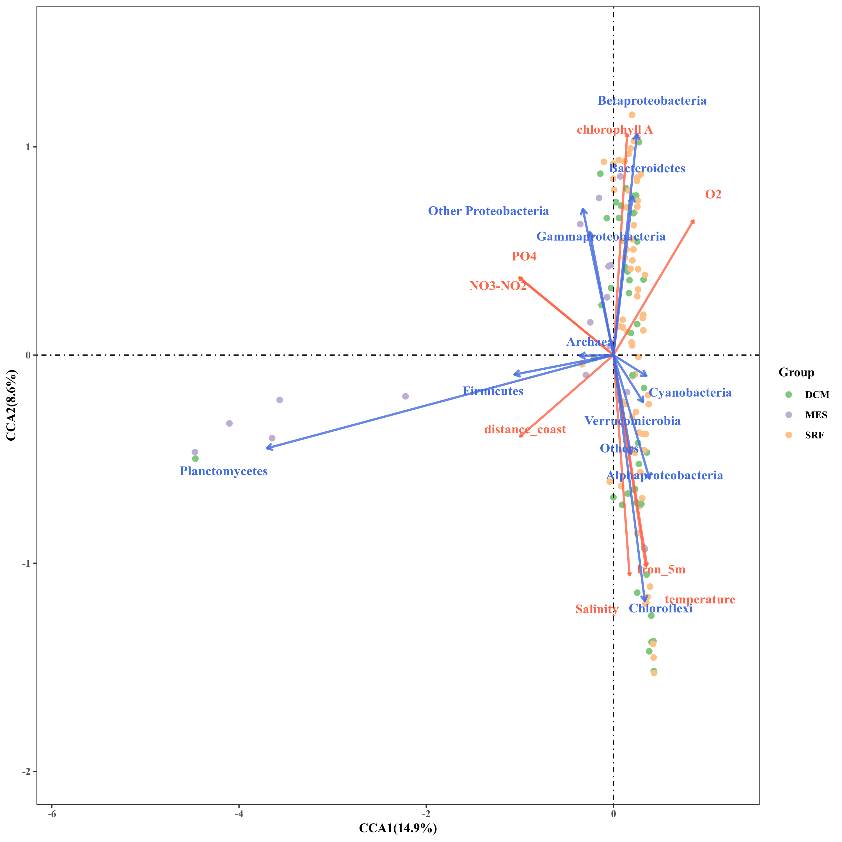


**(B)**


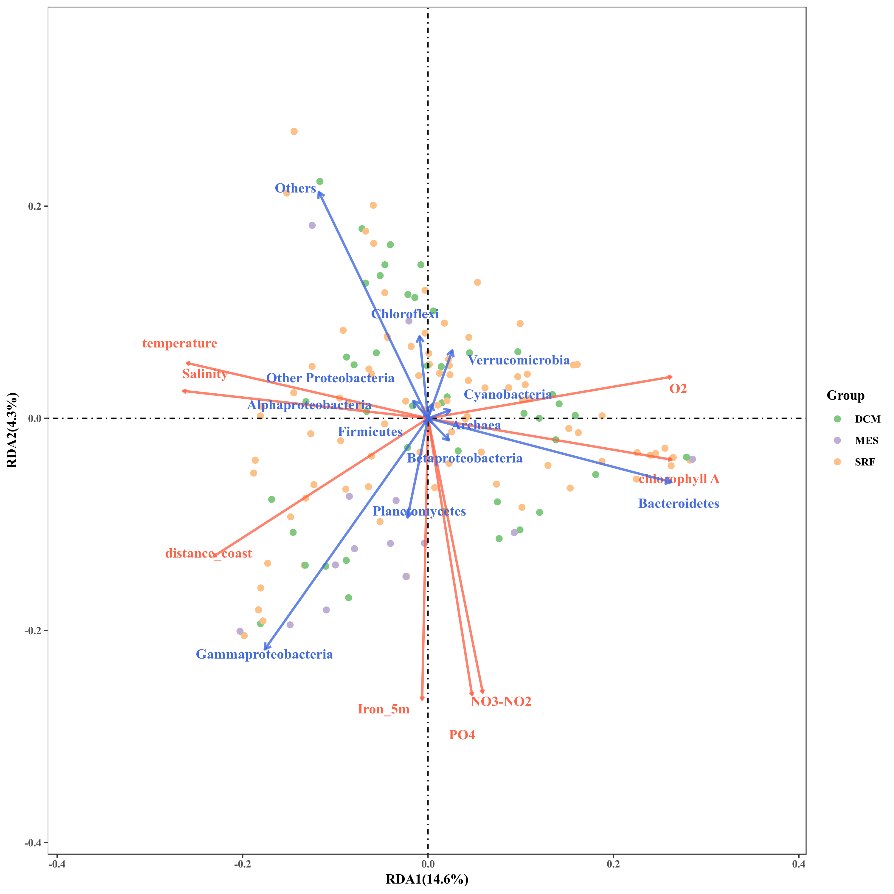


**(A)**


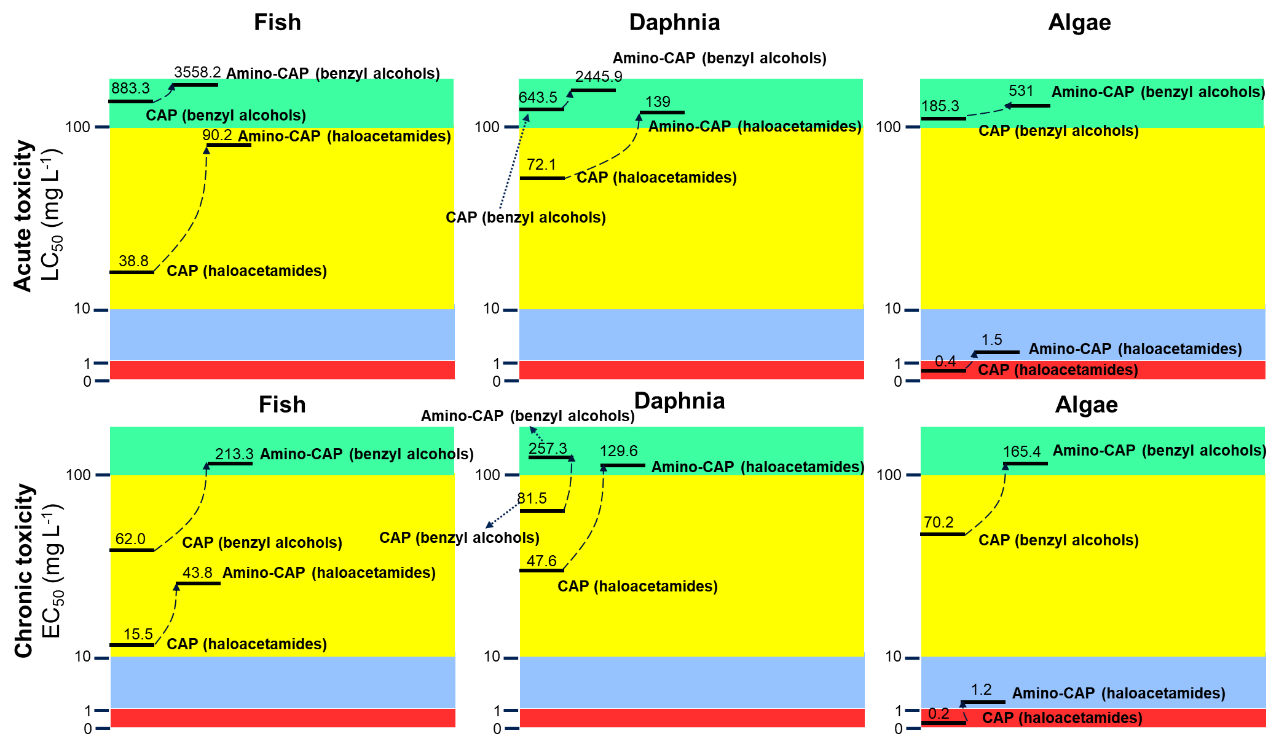


**Supplementary Figure 7.** Toxicity assessment of the chloramphenicol and its degradation intermediate on fish, daphnia, and green algae after degradation using Ecological Structure Activity Relationships (ECOSAR) Program (version 1.11). The toxicity levels of pollutants can be categorized into four levels: “very toxic” with EC_50_ <1 mg L^−1^, which was marked with red color; “toxic” with EC_50_ = 1−10 mg L^−1^ (blue); “harmful” with EC_50_ = 10−100 mg L^−1^, which was marked as yellow; and “non-harmful” with EC_50_ > 100 mg L^−1^ (green color) toward aquatic organisms using the technical guidance document (CEC 1996).

**Supplementary Figure 8.** Growth patterns of *Synechocystis* sp. under different experimental conditions including control (without chloramphenicol exposure), 2 mg L^-1^ chloramphenicol (CAP), 5 mg L^-1^ CAP, NTR treated 2 mg L^-1^ CAP, and NTR treated 5 mg L^-1^ CAP. All experiments have been conducted in triplicates (*N*=3).

References

Commission of the European communities. Technical Guidance Document in Support of Commission Directive 93/67/EEC on Risk Assessment for New Notified Substances and Commission Regulation (EC) No 1488/94 on Risk Assessment for Existing Substances. Part II; Environmental Risk Assessment. Office for Official Publications of the European communities, Luxembourg (1996).
